# Clinical Analysis of the Curative Effect of a Transnasal Ileus Tube in the Treatment of Small Bowel Obstruction Caused by a Phytobezoar

**DOI:** 10.1155/2020/4295024

**Published:** 2020-09-28

**Authors:** Yong-Xu Lin, Sun-Jian Wang, Hui-Shun Liang, Su Lin, Li-Yong Bian, Jian Ding, Dan Li

**Affiliations:** ^1^Department of Gastroenterology, Fujian Medical University Union Hospital, Fuzhou 350001, China; ^2^Department of Radiology, Fujian Medical University Union Hospital, Fuzhou 350001, China; ^3^Liver Research Center, The First Affiliated Hospital of Fujian Medical University, Fuzhou 350005, China; ^4^Department of Radiology, Fujian Provincial Hospital, Fuzhou 350001, China; ^5^Department of Gastroenterology, The First Affiliated Hospital of Fujian Medical University, Fuzhou 350005, China

## Abstract

**Objective:**

To investigate the curative effect of a transnasal ileus tube in the treatment of small bowel obstruction caused by a phytobezoar.

**Methods:**

Seventy-one patients with small bowel obstruction caused by a phytobezoar who underwent treatment in three provincial tertiary grade A hospitals in Fujian Province from March 2011 to February 2020 were included in this study. Patients were divided into the following two groups according to the treatment received: (1) conservative group, comprising patients who received medical conservative treatment, and (2) combined group, including patients who received combined medical conservative treatment and transnasal ileus tube placement. The clinical symptoms, changes in abdominal imaging, tube depth of the first day, reduction of pressure volume on the first day after catheterization, length of hospital stay, and nonsurgical rate were compared between the combined and conservative groups.

**Results:**

There was no significant difference in age, sex, history of previous abdominal surgery and abdominal radiotherapy, symptoms at admission, duration of symptoms before admission, signs at admission, laboratory data, and obstruction position between the combined and conservative groups. There was a statistically significant difference in the nonsurgical rate (19/24 vs. 23/47, *P* = 0.014) between the combined and conservative groups. Logistic analysis showed that the duration of symptoms before admission, albumin level, and use of a transnasal ileus tube might be independent factors affecting the transition to surgery for patients with small bowel obstruction caused by a phytobezoar (*P* < 0.05).

**Conclusion:**

Timely conservative medical treatment with transnasal ileus tube placement can effectively improve the nonsurgical rate of small bowel obstruction caused by a phytobezoar. The duration of symptoms before admission, albumin level, and use of a transnasal ileus tube were closely related to whether patients with small bowel obstruction caused by phytobezoar were transferred to surgery.

## 1. Introduction

The formation of a phytobezoar is often related to the large amount of tannic acid and pectin in the food, which are found in higher concentrations in fruits and vegetables such as persimmons, jujube, and hawthorn. Tannic acid and pectin in food can form a water-insoluble gel-like substance after interacting with gastric acid or other proteins in the gastrointestinal tract, which are called phytobezoars [1]. As the gastrointestinal tract moves, the bezoar can enter the small intestine, causing small intestinal obstructions. The treatment of small bowel obstruction caused by phytobezoars includes conservative and surgical treatments; however, the use of a transnasal ileus tube is a new type of treatment. The traditional gastrointestinal decompression tube can only be placed in the stomach and has no direct suction effect on the contents of the small intestine, which cannot achieve an ideal decompression effect [2]. In contrast, the application of a transnasal ileus tube allows the total decompression of the small intestine above the obstruction plane by directly aspirating and decompressing the gas and effusion at the proximal end of the small intestinal obstruction, which can quickly relieve the symptoms of small intestinal obstruction [3]. However, there are few related studies on the curative effect of the transnasal ileus tube on the treatment of small intestinal obstruction caused by phytobezoars. This study was aimed at analyzing the treatment and prognosis of patients with small bowel obstruction caused by a phytobezoar, at exploring the curative effect of the transnasal ileus tube in treating these cases, and at identifying risk factors affecting the transition to surgery in this patient cohort.

## 2. Materials and Methods

### 2.1. Study Participants

**Figure 1:**
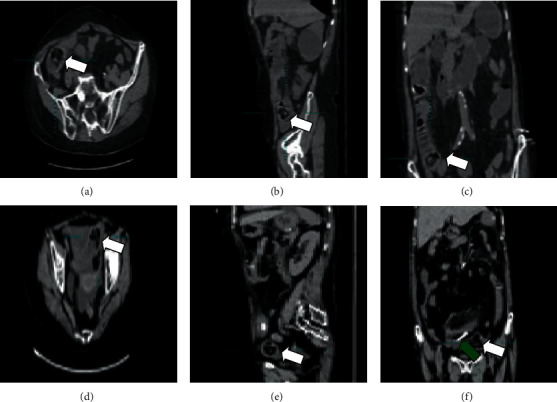
(a–c) Abdominal CT shows an intestinal mass considered to be a phytobezoar in the ileum (white arrow), proximal intestinal dilation, and intestinal effusion. (d–f) The green arrow indicates the transnasal ileus tube placed in the intestinal cavity on the 3rd day; the position of the phytobezoar (white arrow) is lower than previous position; small bowel obstruction is reduced.

Patients with small bowel obstruction caused by phytobezoars who underwent treatment in three provincial tertiary grade A hospitals in Fujian Province from March 2011 to February 2020 were included in this study. The diagnostic criteria were as follows: having clinical symptoms of small intestinal obstruction, such as abdominal pain, abdominal distension, vomiting, and failure of stool and gas pass, and abdominal imaging showing varying degrees of intestinal dilatation and gas-liquid levels, confirmed to be caused by phytobezoars along with medical history (Figures 1(a)–1(c)). The exclusion criteria were as follows: (1) small intestinal obstruction due to other causes (adhesion, hernia, torsion, ischemia, and others); (2) complicated with severe cardiopulmonary dysfunction after admission; (3) incomplete clinical data; and (4) having undergone emergency surgery within 48 hours of admission.

### 2.2. Group

**Figure 2:**
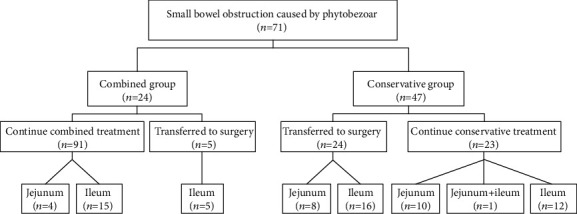
Flowchart of group assignment.

Seventy-one patients were divided into the following two groups according to the different treatment methods: (1) conservative group, comprising patients who received medical conservative treatment for small bowel obstruction caused by a phytobezoar, and (2) combined group, including patients who received the abovementioned conservative treatment combined with transnasal ileus tube application (Figure 2).

### 2.3. Treatment Method

Conservative treatments of small intestinal obstruction caused by a phytobezoar after hospitalization included fasting, gastric tube decompression, enema, intravenous nutrition, and application of prophylactic antibiotics. The transnasal ileus tube was applied in the combined group under radiographic or endoscopic guidance. Patients who were transferred to surgery underwent laparotomy with small intestine incision and stone removal with small intestine decompression.

### 2.4. Observation Index

While monitoring various vital signs and blood biochemical indicators, such as blood routine and electrolyte changes, we recorded the obstruction position, time of alleviation of abdominal distension and pain, defecation and exhaustion recovery time, length of hospital stay, and conversion to surgery in the conservative group, whereas in the combined group, in addition to the abovementioned indicators, the insertion method of the transnasal ileus tube, tube depth on the first day, reduction of pressure volume on the first day after catheterization, time of alleviation of abdominal distension and pain after catheterization, defecation and exhaustion recovery time, and time of alleviation of abnormal abdominal imaging findings were recorded.

### 2.5. Efficacy Judgment

Efficacy judgment was defined as the effectiveness of the treatment, which was classified as healing or improvement. Healing was defined as the disappearance of the patient's clinical symptoms and signs, spontaneous defecation, and complete recovery from exhaustion. The abdominal imaging showed no signs of small bowel obstruction. Improvement was defined as the relief of the patient's clinical symptoms and signs, spontaneous defecation, gradual improvement from exhaustion, and better abdominal imaging findings compared to the findings at admission. Ineffectiveness was defined as nonimprovement or worsening of conditions.

### 2.6. Statistical Method

SPSS version 24.0 (IBM Corp., Armonk, NY) was used for the statistical analyses. Data are expressed as mean ± standard deviation for normally distributed continuous variables and as absolute numbers for categorical variables. For group comparison, Student's *t*-test (for normally distributed variables) was used for continuous variables, and the chi-squared test was used for categorical variables. Univariate analysis was performed using the *t*-test or chi-squared test, whereas binomial logistic regression was used for the multivariate analysis. *P* < 0.05 was considered statistically significant.

## 3. Results

### 3.1. Clinical Characteristics of the Patients in Each Group

Among the 71 patients, 42 were male and 29 were female, with an average age of 62 years (range, 11-88 years). Twenty-four patients received combined treatment, and 47 received conservative treatment alone. There was no significant difference in age, sex, history of previous abdominal surgery and abdominal radiotherapy, symptoms at admission, duration of symptoms before admission, signs at admission, laboratory data, and obstruction position between the combined and conservative groups (*P* > 0.05) (Table 1).

Among all patients, the distributions of previous operations were as follows: 4 cases of appendectomy, 1 case of colorectal surgery, 6 cases of gastric surgery, 2 cases of hepatobiliary surgery, 1 case of small bowel surgery, 2 cases of abdominal hernia surgery, 2 cases of urogenital surgery, and 8 cases of obstetrics and gynecological surgery. Only one case had received abdominal radiotherapy.

### 3.2. Treatment Results of the Combined Group

In the combined group, a transnasal ileus tube was successfully inserted in 24 patients, including 18 patients under radiographic guidance and 6 patients under endoscopic guidance. No patient experienced complications, such as intestinal bleeding, intestinal perforation, and aspiration pneumonia, after successful catheterization. Concurrently, there were no cases of catheter rupture or shedding. The depth of catheterization on the first day under radiographic guidance was significantly higher than that of catheterization under endoscopic guidance (*P* < 0.05) (Table 2). Moreover, there were statistically significant differences in the reduction of pressure volume on the first day after catheterization between the radiographic and endoscopic subgroups (*P* < 0.05) (Table 2). There were no statistically significant differences in the time of alleviation of abdominal distension and pain, defecation and exhaustion recovery time, time of alleviation of abdominal imaging, and length of hospital stay between the radiographic and endoscopic subgroups (*P* > 0.05) (Table 2).

### 3.3. Comparison of the Treatment Effects between the Conservative and Combined Groups

The surgical rate in the combined group was significantly lower than that in the conservative group (5/24 vs. 24/47, *P* = 0.014) (Table 1).

### 3.4. Treatment Results of Patients Who Were Transferred to Surgery

In the conservative group, 24 patients had poor results after conservative treatment and were transferred to surgical treatment. In the combined group, five patients with transnasal ileus tubes under radiographic guidance who showed nonimprovement in symptoms were transferred to surgery. The size of the bezoar in patients who were transferred to surgery was based on the maximum diameter (cm). The maximum diameter ranged from 2.0 to 8.0 cm, with an average of 5.0 cm. The surgical complications of 29 patients undergoing surgery are as follows (Table 3). There were no deaths in patients who were transferred to surgery. All patients were cured, or their symptoms improved.

### 3.5. Univariate Analysis of Factors of Patients Who Were Transferred to Surgery

There were significant differences in the duration of symptoms before admission, albumin level, use of a transnasal ileus tube, and length of hospital stay between patients who were and were not transferred to surgery (*P* < 0.05) (Table 4).

### 3.6. Multivariate Analysis of Factors of Patients Who Were Transferred to Surgery

The different independent factors were entered into a multivariate analysis. The results showed that the duration of symptoms before admission, albumin level, and use of the transnasal ileus tube may be independent factors affecting the transition to surgery for patients with small bowel obstruction caused by phytobezoars (*P* < 0.05) (Table 5).

## 4. Discussion

Apart from indigestible food consumption or presence of foreign objects, many risk factors have also been described that facilitate the development of bezoars, including diabetes mellitus, hypothyroidism, inability to chew food, history of abdominal surgery, gastrointestinal dysfunction, and drugs [4, 5]. Although a bezoar can enter the small intestine, incarceration may occur when the diameter of a bezoar is >2.5 cm, thereby causing small bowel obstruction [6]. It is worth noting that in one of our pediatric cases, small bowel obstruction occurred due to the presence of a 2.0 cm diameter bezoar, indicating that the presence of the obstruction caused by bezoars may be related to individual differences among patients. In this study, most patients (*n* = 49) showed an obstruction located at the ileum, which is consistent with the findings of most reports [7]. The reason may be related to the narrowing of the ileal lumen or the reduction of water content in the ileum [8, 9]. When the small intestine is obstructed, owing to the inability of the intestinal contents to be discharged properly, gas and fluid accumulation occurs, leading to increased pressure of the intestinal cavity, expansion and congestion of the intestinal wall, intestinal mucosal ischemia and necrosis, and changes in intestinal flora structure. If it is not properly and timely managed, it may lead to many complicated and dangerous clinical symptoms [10]. The rapid and effective decompression of the small intestine is the key to the treatment of small intestinal obstruction caused by a phytobezoar. By reducing the gas and liquid accumulated in the gastrointestinal tract, which could reduce intestinal cavity expansion, the blood flow in the intestinal wall will be restored and can help to relieve or cure small bowel obstruction [11–13].

Conservative treatment includes fasting, gastric tube decompression, enema, intravenous nutrition, and application of prophylactic antibiotics, among others. Conservative treatment reduces the risk of surgery. However, the length of the common gastric tube is short and can only reach the stomach. It can only drain out the gas and liquid in the stomach cavity and the upper small intestine. Therefore, the decompression effect is limited; patients often do not achieve adequate decompression and may experience secondary intestinal blood flow disorders, water and electrolyte disorders, and acid-base balance disorders. Therefore, conservative treatment cannot effectively alleviate some low and middle intestinal obstructions in time [11].

The transnasal ileus tube is an important method for the symptomatic treatment of small intestinal obstruction caused by phytobezoars. Its clinical application has gradually expanded, especially in the treatment of low and middle intestinal obstruction. The ileum obstruction caused by phytobezoars can be cured by conservative treatment combined with early placement of a transnasal ileus tube. The transnasal ileus tube can effectively and quickly reach the location of the small intestinal obstruction for negative pressure suctioning, quickly alleviating the abdominal symptoms of patients, reducing the patient's intra-abdominal pressure, improving the blood circulation and edema of the intestinal wall of the obstructed section, reducing the intestinal lesions, and improving the patients' general condition significantly, which is consistent with the findings of most reports [11–13]. In addition, according to the composition of the bezoar, coke, soybean oil, diatrizoate, and others can be injected through the transnasal ileus tube to relieve intestinal obstruction, partly due to dissolving the bezoar [14]. In this study, symptoms of small bowel obstruction in 19 patients were relieved at approximately 2-3 days after transnasal ileus tube placement (Figures 1(d)–1(f)). At present, the insertion methods of the transnasal ileus tube in the clinic mainly include radiographic and endoscopic guidance. The transnasal ileus tube can be placed deeper under radiographic guidance than under endoscopic guidance, which can quickly alleviate the low and middle bowel obstructions. However, for transnasal ileus tubes placed under radiographic guidance, the operative time is longer and there is a risk of gastrointestinal perforation, especially for patients with weak intestinal walls. Operators and patients are exposed to radiation, which is contraindicated for some minority groups, such as pregnant women. Therefore, the insertion method should be selected according to individual differences among the patients, with consideration of the quick and effective delivery of the transnasal ileus tube to the obstruction position to reduce the occurrence of complications, which is of very important clinical significance. After the tube is placed, the gas volume drained should be recorded in detail because it is clinically more important than the liquid drained for the relief of small bowel obstruction. Concurrently, the patient's signs should be closely monitored after the insertion of the tube, and we should confirm whether the obstruction is cleared in time by reexamination of abdominal CT. In this study, five patients in the combined group who were transferred to surgery had symptoms before admission for a long time; hence, the time from onset of small bowel obstruction to tube insertion was too long. Thus, we considered whether the transfer to surgery is related to the excessive intestinal wall ischemia. This study shows that the duration of symptoms before admission, albumin level, and use of the transnasal ileus tube may be independent predictors of whether patients with small bowel obstruction caused by a phytobezoar will be transferred to surgery. Therefore, in patients with low albumin concentration, large diameter of the bezoar, longer obstruction time, longer duration of intestinal wall ischemia, and presence of strangulated intestinal obstructions, timely transnasal ileus tube application or surgical treatment is a safer choice.

Surgery is still an effective method for managing small intestinal obstruction caused by a phytobezoar. In this study, 29 patients underwent surgical treatment for small bowel obstruction. The currently used surgical methods mainly include laparotomy with small intestine incision and stone removal with small intestine decompression. It is worth noting that there may be multiple bezoars. In this study, one patient had to undergo secondary operation because not all bezoars were detected preoperatively or during the first operation. There are certain risks of surgical treatment, including the risk of anesthesia and possible postoperative complications, such as aspiration pneumonia, incision infection, exacerbation of cardiovascular and cerebrovascular diseases, abdominal infection, and intestinal fistula. Especially for elderly patients with small bowel obstruction caused by a phytobezoar, surgery remains quite challenging. Therefore, it is important to fully evaluate and explore all bezoars before and during the operation to avoid secondary surgery. In addition, whether the size and quantity of bezoars can be measured by abdominal imaging to determine whether the patients need to undergo surgery is worth exploring.

The main limitation of this study is its retrospective design, which may have led to selection bias. Simultaneously, given that small intestinal obstructions caused by a phytobezoar are not common in clinical settings, some factors that may affect patients' transition to surgery have not been included; thus, more samples must be accumulated. In the future, we will attempt to identify more effective drugs to dissolve bezoars and will augment auxiliary equipment in addition to the transnasal ileum tube to achieve the purpose of mechanical lithotripsy.

## 5. Conclusions

In conclusion, the transnasal ileus tube has its unique advantages in the treatment of small intestinal obstruction caused by a phytobezoar. In particular, selecting an appropriate tube insertion method according to the individual differences of patients and placing the tube successfully as soon as possible have very important clinical significance for the prognosis of patients.

## Figures and Tables

**Table 1:** Clinical findings of the combined and conservative groups.

Clinical findings	Combined group (*n* = 24)	Conservative group (*n* = 47)	*P*
Age (years)	66.5 ± 18.3	59.6 ± 19.5	0.158
Sex (male/female)	15/9	27/20	0.682
History of previous abdominal surgery [*n* (%)]	10 (41.7)	15 (31.9)	0.416
History of previous abdominal radiotherapy [*n* (%)]	1 (4.2)	0	0.159
Symptoms [*n* (%)]			
Abdominal pain	24 (100)	46 (97.9)	0.472
Abdominal distension	19 (79.2)	40 (85.1)	0.528
Failure of stool	23 (95.8)	45 (95.7)	0.986
Failure of gas pass	23 (95.8)	43 (91.5)	0.499
Nausea	13 (54.2)	27 (57.4)	0.792
Vomiting	19 (79.2)	33 (70.2)	0.420
Duration of symptoms before admission (days)	6.7 ± 5.6	7.7 ± 5.2	0.406
Signs [*n* (%)]			
Temperature (>37.5°C)	0	3 (6.4)	0.206
Heart rate (>100 beats/min)	1 (4.2)	1 (2.1)	0.623
Bowel sounds (<3 times/min)	10 (41.7)	19 (40.4)	0.920
Acute peritonitis	0	2 (4.3)	0.305
Laboratory data			
WBC count (×10^9^/L)	9.5 ± 3.8	9.4 ± 3.5	0.860
Hemoglobin (g/L)	122.0 ± 23.9	126.7 ± 17.0	0.392
Albumin (g/L)	32.8 ± 4.4	33.9 ± 5.2	0.382
Potassium (mmol/L)	4.0 ± 0.6	4.0 ± 0.6	0.911
Sodium (mmol/L)	137.6 ± 4.1	137.0 ± 3.6	0.494
Obstruction position			
Jejunum/ileum/jejunum+ileum	4/20/0	18/28/1	0.120
Effectiveness of nonsurgical therapy [*n* (%)]	19 (79.2)	23 (48.9)	0.014

^∗^Except if otherwise noted, values are presented as mean ± standard deviation or number (%).

**Table 2:** Comparison of the insertion methods of the transnasal ileus tube.

Insertion method	Radiography (*n* = 13)	Endoscopy (*n* = 6)	*P*
Tube depth of the first day (cm)	268.0 ± 40.8	97.5 ± 6.5	0.001
Reduced of pressure volume at the first day (mL)	484.6 ± 395.2	117.0 ± 135.6	0.008
Alleviation of abdominal distension and pain (days)	1.9 ± 1.0	3.2 ± 2.3	0.109
Defecation and exhaust recovery time (days)	2.7 ± 2.4	5.0 ± 2.4	0.064
Alleviation of abdominal imaging (days)	3.6 ± 1.4	5.3 ± 3.1	0.144
Length of hospital stay (days)	9.3 ± 4.0	11.7 ± 4.1	0.250

^∗^Values are presented as mean ± standard deviation.

**Table 3:** Complications after surgery.

Complications	Number (*n*)
Secondary surgery	1
Incision site infection	1
Incision site infection+abdominal infection+intestinal fistula	1
Acute myocardial infarction	1
Aspiration pneumonia	1

**Table 4:** Univariate analysis of factors of patients' transition to surgery.

	Surgery (*n* = 29)	No surgery (*n* = 42)	*P*
Age (years)	57.3 ± 22.0	65.1 ± 16.7	0.092
Sex (male/female)	20/9	22/20	0.162
History of previous abdominal surgery [*n* (%)]	9 (31.0)	16 (38.1)	0.540
History of previous abdominal radiotherapy [*n* (%)]	1 (3.4)	0	0.226
Symptoms [*n* (%)]			
Abdominal pain	29 (100)	41 (97.6)	0.403
Abdominal distension	27 (93.1)	32 (76.2)	0.062
Failure of stool	27 (93.1)	41 (97.6)	0.353
Failure of gas pass	26 (89.7)	40 (95.2)	0.366
Nausea	19 (65.5)	21 (50.0)	0.195
Vomiting	24 (82.8)	28 (66.7)	0.132
Duration of symptoms before admission (days)	9.9 ± 6.4	5.7 ± 3.5	0.001
Signs [*n* (%)]			
Temperature (>37.5°C)	2 (6.9)	1 (2.4)	0.353
Heart rate (>100 beats/min)	0	2 (4.8)	0.233
Bowel sounds (<3 times/min)	14 (48.3)	15 (35.7)	0.290
Acute peritonitis	2 (6.9)	0	0.084
Laboratory data			
WBC count (×10^9^/L)	10.1 ± 3.7	9.0 ± 3.4	0.215
Hemoglobin (g/L)	124.0 ± 18.4	125.7 ± 20.7	0.740
Albumin (g/L)	31.5 ± 4.5	34.9 ± 4.9	0.005
Potassium (mmol/L)	4.0 ± 0.7	4.0 ± 0.5	0.803
Sodium (mmol/L)	136.7 ± 4.0	137.6 ± 3.7	0.344
Obstruction position			
Jejunum/ileum/jejunum+ileum	8/21/0	14/1/27	0.594
Receive transnasal ileus tube treatment	5 (17.2)	19 (45.2)	0.014
Length of hospital stay (days)	16.3 ± 7.6	9.3 ± 3.7	0.001

^∗^Except for special instructions, values are presented as mean ± standard deviation or number (%).

**Table 5:** Logistics analysis of factors of patients' transition to surgery.

Factors	*B*	SE	Wald	*P*	OR	95% CI
Duration of symptoms before admission (days)	0.151	0.065	5.349	0.021	1.162	1.023–1.321
Albumin (g/L)	-0.139	0.068	4.232	0.040	0.870	0.762–0.993
Receive transnasal ileus tube treatment	-1.869	0.716	6.822	0.009	0.154	0.038–0.627

## Data Availability

The datasets generated/analyzed during the present study are available from the corresponding authors on reasonable request.
